# Draft Genome Sequence of *Pandoraea* sp. Strain SD6-2, Isolated from Lindane-Contaminated Australian Soil

**DOI:** 10.1128/genomeA.00415-13

**Published:** 2013-07-05

**Authors:** Hafizah Pushiri, Stephen L. Pearce, John G. Oakeshott, Robyn J. Russell, Gunjan Pandey

**Affiliations:** CSIRO Ecosystem Sciences, Acton, ACT, Australiaa; Research School of Chemistry, Australian National University, ACT, Australiab; Faculty of Environmental Studies, Universiti Putra Malaysia, Serdang, Malaysiac

## Abstract

*Pandoraea* sp. strain SD6-2 is a δ-hexachlorocyclohexane-degrading bacterial strain isolated from lindane-contaminated soil in Queensland, Australia. The genome of SD6-2 was sequenced to investigate its ability to degrade δ-hexachlorocyclohexane. Here we report the annotated genome sequence of this strain.

## GENOME ANNOUNCEMENT

Strain SD6-2 was isolated during a hexachlorocyclohexane (HCH) degradation study in Australia. Among several other strains from soil from Queensland, Australia, contaminated with γ-HCH (also known as the insecticide lindane), this strain was isolated after six cycles of enrichment using 50 ppm δ-HCH as the sole carbon source. Degradation studies using this strain revealed that it partially degrades δ-HCH, although very slowly compared to other known HCH degraders (1). However, given its superior degradation activity compared to other isolates in the study, strain SD6-2 was chosen for genome sequencing.

Genomic DNA of SD6-2 was prepared using the Qiagen Genomic-tip 20/G kit for bacteria, following the manufacturer’s instructions. We sequenced 500-bp fragments using Illumina HiSeq2000 technology at the John Curtin School of Medical Research, Australian National University. The Ray assembler was used to assemble 13,475,774 100-bp paired-end reads using a k-mer length of 63 (2). This assembly generated 51 contigs >500 bp in length, with an *N*_50_ value of 198,404 bp. The total size of the assembly was 5.7 Mb, with a GC content of 62.5%. Paired-end information combined 21 of the contigs into 7 scaffolds, making a total of 37 scaffolds or contigs for the assembly.

Annotation of the genome using the NCBI Prokaryotic Genomes Automatic Annotation Pipeline (PGAAP) predicted 5,148 protein-coding sequences in SD6-2, of which 1,222 (23.7%) were hypothetical proteins. The current assembly was also predicted to contain 67 tRNA sequences and 4 rRNA clusters. A BLAST search using the 16S rRNA sequence of SD6-2 against the NCBI database revealed 99.8% (1,405-nucleotide [nt] alignment) identity between SD6-2 and *Pandoraea* sp. The genome size and GC% of SD6-2 are consistent with the only other sequenced *Pandoraea* genome (*Pandoraea* sp. B-6, GenBank accession number AKXS00000000); however, further investigation is needed to determine the true identity of SD6-2.

To the best of our knowledge, all currently characterized HCH degraders have been found to degrade HCH via the same degradation pathway, which requires the *linA-linF* genes (3–6). A BLAST search within the SD6-2 genome using the *linA-linF* protein sequences (YP_003545302, BAI96793, YP_003544005, YP_003547114, YP_003547119, and BAI98845) of *Sphingobium japonicum* UT26S from the NCBI database found none of these genes or their homologues in the SD6-2 genome (1). At this stage, strain SD6-2 was again tested for δ-HCH degradation but no degradation activity was observed (S. Pearce, unpublished data). This result, together with the association of the *lin* genes with IS*6100* elements (5, 7, 8), may explain the loss of degradation activity since the first degradation assay.

### Nucleotide sequence accession numbers.

This whole-genome shotgun project has been deposited at DDBJ/EMBL/GenBank under the accession number AQOU00000000. The version described in this paper is version AQOU01000000.
